# Malawian fathers’ views and experiences of attending the birth of their children: a qualitative study

**DOI:** 10.1186/1471-2393-12-141

**Published:** 2012-12-05

**Authors:** Lucy Ida Kululanga, Address Malata, Ellen Chirwa, Johanne Sundby

**Affiliations:** 1 Department of Community and Mental Health, Kamuzu College of Nursing, University of Malawi, P.O. Box 415, Blantyre, Malawi; 2 Department of Maternal and Child Health, Kamuzu College of Nursing, University of Malawi, Private Bag 1, Lilongwe, Malawi; 3 Department of Community Medicine, Institute of Health and Society, Faculty of Medicine, University of Oslo, P.O. Box 1130, Blindern, Oslo, 0318, Norway

**Keywords:** Male partner, Childbirth, Support, Labour, Malawi

## Abstract

**Background:**

Exploring the experiences and views of men who had attended the birth of their children is very vital, especially in a setting where traditionally only women accord women support during labour and childbirth. The insights drawn from the male partners’ views and experiences could enhance the current woman-centred midwifery model that encompasses the needs of the baby, the woman’s family and other people important to the woman, as defined and negotiated by the woman herself. This paper explored the views and experiences of men who attended the birth of their children from two private hospitals in an urban setting in southern Malawi.

**Methods:**

This study used an exploratory descriptive qualitative approach. The data were collected through in-depth interviews from 20 men from Blantyre, a city in the southern part of Malawi, who consented to participate in the study. These men attended the birth of their children at Blantyre Adventist and Mlambe Mission Hospitals within the past two years prior to data collection in August 2010. A semi-structure interview guide was used to collect data. Qualitative content analysis was used to analyse the data set.

**Results:**

Four themes were identified to explain the experiences and views of men about attending childbirth. The themes were motivation; positive experiences; negative experiences; reflection and resolutions. The negative experiences had four sub-themes namely shame and embarrassment, helplessness and unprepared, health care provider – male partner tension, and exclusion from decision-making process.

**Conclusions:**

The findings showed that with proper motivational information, enabling environment, positive midwives’ attitude and spouse willingness, it is possible to involve male partners during childbirth in Malawi. Midwives, women and male peers are vital in the promotion of male involvement during childbirth. In addition, midwives have a duty to ensure that men are well prepared for the labour and childbirth processes for the experience to be a positive one.

## Background

Male partners’ attendance at birth was still controversial in the United Kingdom even as late as the 1970s [1] and in the United States of America until 1947 when Dr. Robert Bradley advocated for continual presence of the father in labour and birth as labour coaches [2]. Later, attempts were made by advocates of natural childbirth in the United States of America to involve fathers in the process of childbirth in order to promote paternal bonding [3]. Unfortunately, such prominent advocates of the natural childbirth movement such as Odent did not place great emphasis on paternal participation during birth. For example, Odent discouraged the presence of male partners for fear of distracting women from the natural process of labour [4]. However, male partner’s presence at childbirth is now almost universal in industrialised countries [5]. For instance, in the United States in the 1980s at least 80% of the fathers regardless of marital status were permitted by health professionals to be present during labour and birth [6].

A number of factors have influenced the high participation rate of male partners during childbirth in the west. Wertz and Wertz argued that, during the post-war era, a concept of ‘family togetherness’ was promoted throughout American society [7]. The admission of the male partners into the labour room was constructed as a public statement of family togetherness, a sign of a strong middle-class marriage [7]. Equally, male partner’s presence during childbirth was construed as a shared experience for couples [8]. Therefore, male partner’s attendance at birth was associated with a high-level quality of intimate relationship between a man and a woman. In addition, male partner attendance and active participation at labour and childbirth was associated with a belief of a ‘good’ partner, ‘good’ father [7], and a public statement of the strength of the father-infant bond [9].

Seel argued that the changing family and social patterns was one of the reasons for male partner’s presence in the labour ward [10]. He further argued that as people moved away from the place where they were brought up and set up homes in new areas, they lost close**-**knit networks. Thus, couples with loosely-knit networks were more likely to do things together and to turn to each other for comfort and support. In view of this, women sought the support of their male partners during labour and childbirth [10].

There are wide cultural variations worldwide pertaining to childbirth and male involvement. Birthing in Nepal, especially among Bajura people, is considered to be ‘polluted’. As a result, the woman is required to labour and birth alone, not even family members are allowed to be present and/or touch the woman. Solitary confinement continues for a number of weeks after birth [11]. Similarly, a Jewish birthing woman is considered ‘polluted’ according to Rabbinic law [12]. As such, a male partner is not allowed to be present during childbirth to avoid being contaminated. Conversely, a Siriono Bolivian male partner is expected to be present during childbirth in order to cut the cord, which is a way of claiming paternity [13], p. 15.

Studies undertaken to explore views and experiences of men during childbirth have reported a wide range of experiences that include improvements in the couple’s positive attitude towards the birthing process [14], enhanced father-baby attachment, and father’s increased participation in early caretaking activities [15]. Pride related to fatherhood, increased respect for women [5] and improved partners’ relationships [16] were reported. On the other hand, some studies have documented less favourable aspects of male partners’ presence at childbirth. For instance, hidden fears, dissatisfaction with their own ability to support their partners [5], more tension and excitement during birth and inability to cope with partner’s pain in labour [17].

Traditionally, in Malawi as in other African settings, women have been attended and supported by other women during labour and birth. In a study done in rural communities of Mwanza district in southern Malawi, Kululanga et al. [18,19] documented that childbirth was perceived as a women’s issue, a territory where women would not want men to invade. The concept of male partner attendance at labour and birth is perceived as a foreign culture, not commonly practiced in Malawi [18,19]. In addition to tradition, another barrier to male partner’s attendance at childbirth is infrastructure. Most of the public health facilities do not provide privacy for labouring women and the presence of someone’s male partner would breach another labouring woman’s privacy [18,20].

Previous studies have reported the beneficial effects of male partner involvement in the birthing process, however, the results may not be observed in Malawi because these studies were conducted in industrialized countries. Published study findings on men’s childbirth attendance in Malawi or countries comparable to Malawi are scarce. Therefore, the aim of this study was to explore the views and experiences of men who had attended the birth of their children.

## Methods

### Design

The design of the study was an exploratory descriptive approach that utilized qualitative methods. The data were collected using in-depth interviews.

### Settings

Data for this study were collected from men whose partners gave birth to their babies at Blantyre Adventist and Mlambe Mission hospitals in Blantyre city, Malawi. The two hospitals were purposively chosen because they allowed male partner’s presence during labour and birth.

### Blantyre Adventist Hospital

Blantyre Adventist Hospital (BAH) is located at the centre of Blantyre city in southern Malawi. The American missionary doctors of the Seventh-Day Adventist (SDA) Church established the hospital in 1957. It is a forty-bed private-for-profit hospital. Although the hospital belongs to the SDA church, it is completely self-financed, receiving no church subsidies. Almost all of the clients of BAH have an insurance medical scheme, pay cash, or are part of a firm or organization that covers the account on their behalf.

The BAH offers specialized obstetric care operated by an obstetrician, state registered nurse/midwives (SRNMs) and nurse/midwives technicians (NMTs). Maternal care services that are offered at the hospital include antenatal care that includes ultra-sound scanning, childbirth classes, labour and childbirth services, postnatal care of mother and baby, 2 weeks post-normal birth and 1-week post caesarean section. Antenatal and postnatal care services are offered by an obstetrician at the outpatient department. Clients have to book an appointment in order to be seen by their obstetrician. The number of antenatal care visits depends on individual obstetric needs. Optional childbirth classes are also offered by a midwife at the outpatient department.

During childbirth, the clients choose whom they would want to assist the birth of their baby, a midwife, a general doctor or an obstetrician. The cost of childbirth depends on who conducts the birth and the mode of birthing, for example, normal or caesarean section. The cost of childbirth conducted by a midwife is lower than a birthing conducted by an obstetrician.

### Mlambe Mission Hospital

Mlambe Mission Hospital (MMH) is situated 30 kilometres north of Blantyre city. It is a 254-bed facility that is run by the Roman Catholic Church. The hospital is one of the Christian Hospital Association of Malawi (CHAM) facilities. CHAM is an ecumenical, not-for-profit non-governmental umbrella organization of Christian-owned health facilities. CHAM offers about 37% of health care services in Malawi [21]. Ninety percent of CHAM health facilities are located in the rural settings of the country where, in most cases, there are no government facilities. Therefore, through Service Level Agreement (SLA) CHAM signed contract with Ministry of Health (MoH) to provide free maternal health care. Mlambe Mission Hospital signed a SLA contract with Blantyre District Health Office that enables it provide maternal health care services to the people around its catchment area. In addition, the hospital receives obstetric referrals from six government health centres. The hospital offers antenatal care (ANC), labour, birth, and postnatal care services. An obstetrician, general practitioners, clinical officers, SRNMs and NMTs offer the MCH services.

Mlambe Mission Hospital offers two levels of maternity care services, low cost care and private care. The low-cost maternity care services cater to poor women who cannot access government hospitals. The private maternity care services are similar to that of BAH except for the childbirth classes. However, the cost of private maternity care at MMH is lower than that of BAH because of the financial support from the Roman Catholic Church and CHAM. For instance, a normal vaginal birth would cost around MWK 100,000, an equivalent of 333USD, and a caesarean section would cost MWK 250, 000 (833USD) at BAH. At MMH, a normal birth costs around MWK 50,000.00 (166USD) and caesarean section costs around MWK 100,000 (333USD). However, in the government hospitals these services are free.

### Participants and recruitment

Twenty indigenous Malawian men were purposively selected to participate in this study. Purposive sampling strategy was used because the participants could provide relevant, insightful information on the phenomenon being explored. In addition, variation in sample with regard to educational background, age, social economic status and parity was sought in order to include a range of perspectives. Some of the participants were recruited from the health facilities as they came with their female partners for postnatal check up or family planning services, while other men were recruited through the snowball technique. Snowball sampling, a procedure that relies on referrals from initial informants to generate additional participants who met the eligibility criteria for the study, but are hard-to-reach population [22]. Therefore, men who had been interviewed were asked if they knew other men who attended their children’s birth. The participants represented urban men who were present when their female partners gave birth to their children and the women gave birth at BAH and MMH. In addition, the participants’ infants were 2 years and under at the time of inclusion to the study.

Twenty interviews were conducted and were sufficient to achieve data saturation. Green and Thorogood stated that “the experience of most qualitative researchers is that in interview studies little that is ‘new’ comes out of transcripts after you have interviewed 20 or so people” [23], p. 120. Bowen described data saturation as bringing new participants continually into the study until the data set is complete, as indicated by data replication or redundancy [24].

### Ethical consideration

Permission to conduct the study was obtained from Malawi College of Medicine Research and Ethical Committee (protocol number P.05/10/948) and the Regional Committee for Medical and Health Research Ethics in Norway (vår ref. 2009/968a). Permission to access participants was sought from the directors and chief nursing officers of BAH and MMH. All participants were informed about the purpose of the study. The men were also informed that their participation was voluntary and that they were free to withdraw from the interview and the study at any time without giving a reason. The men were further informed that their withdrawal would not affect their entitlements to health services. A written informed consent was obtained from individual participants.

### Data collection

Data were elicited between August 2010 and January 2011 in the city of Blantyre. A semi-structured interview guide was administered to 20 individuals that consented to participating in the study. The structured part included participants’ demographic data and the open-ended part captured qualitative data. The semi-structured interview guide was developed basing on a literature review. The questions in the interview guide were broad and open-ended that allowed for both directed questions and freer exploration of unanticipated issues raised by the participants. See Additional file 1: Appendix 1 for the interview guide. The interviews were conducted in Chichewa and lasted between 40 to 60 minutes. The health facility management provided a private office to carry out interviews for participants who opted to be interviewed at the health facility. Ten men were interviewed at the health facilities and six at their place of work. The four remaining participants were interviewed in their respective homes. All interviews were audio-recorded. Hand written notes were taken during the interviews and later expanded into transcripts. At the end of each interview, a summary of the notes were read to the participant in order to verify the data. The participants were given a soft drink and a snack after the interview as a gesture of appreciation.

### Data analysis

Data analysis was undertaken simultaneously with data collection in order to identify new and important issues that could be addressed during the subsequent interviews. The taped information was transcribed verbatim and translated from vernacular language into English. Observational field notes were incorporated into the data for analysis. General principles of qualitative content analysis by Graneheim and Lundman guided data analysis [25]. The transcribed data were entered in Nvivo 9, software for data storage and management system. The transcripts were read repeatedly and words with similar meanings were grouped into categories. Similar categories were grouped into themes and sub-themes that are presented as findings in this paper. The findings contain direct quotes from participants and the narrations are reported as they were spoken by participants without editing the grammar to avoid losing meaning. Expressions in vernacular language are presented in parentheses and fictitious names are used in the quotes to maintain anonymity of the participants.

### Trustworthiness

The process of data verification was carried out according to Lincoln and Guba’s criteria of rigour in qualitative research that includes credibility, transferability, dependability and confirmability [26], 300. They defined credibility as internal validity and relates to “how vivid and faithful the description of the phenomenon is” [27]. In this study, credibility was enhanced by using participants’ actual words in the report. Since the interviews were done in Chichewa, a vernacular language, and translated into English, there was a potential for misrepresenting a participant’s intended meaning of a word. Therefore, the words were supported by quotations from the interviews. In addition, the investigator had frequent discussion sessions with co-authors and impartial colleagues experienced in qualitative methods, which provided a platform for developing ideas and interpretations that represent the correct picture of reality about the phenomenon under study. Transferability corresponds to external validity and the probability that the research findings can be used in other contexts [28], p.316. In this study, the background and methods used were described as much in detail as possible in order to allow readers to assess the applicability in other contexts.

Dependability corresponds to reliability of the findings and it occurs when another researcher is able to follow the methods and draw similar conclusions to the original research findings [27]. Dependability requires that the argument is complete, allowing the reader to follow and understand it logically without unexplained leaps from argument to conclusion [26], p.300. In this study, dependability was achieved by the involvement of the experienced researchers (co-authors) in qualitative methods, who followed through the progression of the study. Their independent analysis and evaluation of decisions made and consensus, determined whether comparable conclusions could be reached given the same data and research context. Confirmability is described as objectivity whereby the findings of the study represent the results of the inquiry and not the researcher’s biases [29]. Confirmability was achieved through an audit trail constructed through memos and field notes. The notes allowed the investigator and the co-authors to trace the course of the research systematically.

## Results

### Demographic characteristics of the participants

All the participants were married and their ages ranged from 29 to 50 years with an average of 35 years. Table 1 presents demographic characteristics of the participants.

**Table 1:** Demographic characteristics of the participants

**Demographic characteristic**	**Number**
Educational level completed	
Primary level	0
Secondary level	4
Tertiary level	16
Employment	
Paid up jobs	17
Self-employed	3
Ethnicity	
Chewa	6
Ngoni	4
Tumbuka	4
Mang’anja	2
Yao	2
Lomwe	2
Parity of spouse	
First time fathers	9
Second and more time fathers	11
Spouses’ mode of birth	
Normal vaginal birth	16
Caesarean section	4

### Themes

Men’s experiences and views have been categorized into four themes, namely motivation, positive experiences, negative experiences and a period of reflection and resolutions. The negative experience had four sub-themes namely shame and embarrassment, helplessness and unprepared, health care provider – male partner tension, and exclusion from decision-making process.

#### Motivation

Several factors motivated the men to attend the birth of their children. The majority of the men indicated that the midwives informed them during the antenatal period about the male partner’s right to be present at childbirth. Some of the men were requested by their spouses to attend the childbirth while others were encouraged by their peers. The men declared that they had to discuss the issue with their partners and made a joint decision on his attendance at birth. Some of the men attended the childbirth out of curiosity, as expressed in the response below:

"“You know what, I had not seen a woman giving birth and I was curious to see. I had seen pictures in biology books but this time I wanted to have a real experience” (35-year old lawyer)"

#### Positive experiences

The fathers’ attendance at childbirth seemed to have positive impact on the men. For instance, the attendance increased their knowledge about the birthing process and accorded them a chance to be the first persons to welcome their children. Consequently, most of the men added that the knowledge they had acquired through observing the birthing process would enable them to provide support to their partners in future childbirths. The men described how they had acted as advocates for their partners by negotiating for something to be done when their partners were in great pain and in addition to providing the necessary psychological support. Some men said that it was a feeling of reality to witness that the baby had arrived. First time fathers further stated that the birth of their babies changed their social status immediately to that of a father. The midwives started calling them ‘*bambo a mwana*’ meaning the father to the baby.

"“I was overwhelmed with joy to see our new born baby. It was amazing that at the same time all the pain my partner was experiencing ceased” (29 year--old, stock controller)"

#### Negative experiences

"Shame and embarrassment"

Men were asked to describe negative incidents that they encountered during labour and childbirth process. Commonly, all the men stated they felt uncomfortable and somehow distressed during some of the routine procedures undertaken during their partner’s labour and birth, particularly the vaginal examinations. While the men realized that these were necessary to assess the progress of labour, they nonetheless felt ashamed and embarrassed. One man said:

"“The vaginal examinations that the midwives did on my partner put me off. Much as it is a procedure to monitor progress of labour, it was an invasion of our privacy.” (45-year-old, engineer)"

Observing a labouring partner in severe pain was an experience that most men could not easily tolerate. Feelings of fear, anger and frustration were expressed. The men indicated that they feared for the life of their partners and could not imagine the intensity of the labour pains. They also said that they became angry with the midwives after noticing that they were not doing much to relieve the pain. Some of the men were also angry with themselves after knowing that they had no control over the situation. One of them explained that his partner had prolonged labour and she was in great pain but the doctor on call delayed to attend to her. The midwife explained to them that labour was not progressing well and that she had called a doctor on call to come and review the partner. The participant further explained that the midwife anticipated that the partner might go for caesarean section but the doctor’s delay created an intense atmosphere in the labour ward such that the male partner was frustrated. The man expressed his frustrations as follows:

"“It was so frustrating to watch my partner in great pains and not being able to do anything. I was so angry that I found it difficult to control myself. I left the hospital and went to my office.” (35-year-old lawyer)"

The male partners also feared for the lives of their partners. Apparently, the fears were more related to the mode of birth than the childbirth process itself. The men whose partners’ underwent a normal vaginal birth experienced negative emotions when their partners were having severe labour pains and became restless. Fear of losing a partner also set in when the men saw the amount of blood that the partners had lost during birth. Most of the men stated that they were terrified with the amount of blood. Most of them pretended to be strong for their partners but were in fact afraid. However, positive emotions were restored when the baby was born, the bleeding was controlled, and the mess around were cleaned up, as expressed by one of the participants below:

"“I didn’t know that women bleed so much when they are giving birth. My partner bled so much and I was so scared that I was going to lose her.However, the bleeding stopped when she was given an injection and the placenta was removed.” (30-year- old stock controller)."

Similarly, four men who attended elective caesarean section procedures expressed feelings of fear for the outcome of the operation (birth). Most of them could not endure looking at a partner being cut open and seeing her abdominal contents. They indicated that it was a frightening experience to see the incision, the instruments used on the partner and the blood that spilled. One participant stated that:

"“I was allowed in the operating theatre to be with my partner and observe the operation. It was the first time to enter the operating theatre. I was a bit scared. They gave my partner an injection on the spine and numbed her. She was talking to me but could not feel pain. The moment they cut her abdomen, I fainted. I realized after sometime that I was ushered into the recovery room by one of the midwives.”(39-year-old programme coordinator)"

The four men who attended the caesarean section of their wives declared that they would not attend future childbirth while those who attend the normal vaginal birth indicated that they would consider attending in the future.

"Helplessness and unprepared"

An inability to offer physical support to a partner was felt by most of the men as a negative experience. Most of the men expressed that they lacked knowledge and skill to offer physical support. They stated that they did not attend childbirth classes and did not know much about physical support. Lack of knowledge frustrated most of the men, as they did not understand what was going on and how to assist their partners through labour. The men felt that attending antenatal care sessions with their partners did not prepare them for labour and birth and their role in these processes. They stated that antenatal care services concentrated more on the wellbeing of the mother and foetus and very little information was given to the male partners about birth preparedness and no information was provided on the childbirth process or male partner’s role.

"“I didn’t know what to do with my partner when she was restless and in pain. I felt my presence was useless as I failed to assist her when she needed help.”(40-year-old accountant) "

"Health worker – male partner tension"

Few men also indicated that their presence in the labour ward created some tension between them and the health care providers. Some men perceived that they were labelled ‘difficult’ because they kept on asking for explanation as to what was happening to their partners, and even demanding for drugs when their partners were in severe pain. Other male partners avoided confrontation with the health professionals for fear that their actions might affect negatively the care of their partner.

"“I avoided to ask so many questions and to demand for care because I felt that my partner might be neglected. Although at times I felt that my partner deserved more that what she was getting in terms of medical care.” (30-year-old businessman)"

"Exclusion from decision-making process"

All the participants expressed that neither they nor their partners were involved in decision making about the obstetric care given. The medical professionals made all the decisions and the couples were just informed of what they were expected to do. This practice did not bother most of the men because they felt that they came to the hospital to seek medical care from the medical professionals who were the experts. The couple were passive recipients of care. However, few of the participants felt that the medical professionals needed to consult the couple regarding the care.

"“We were not involved in any decisions that were being made regarding my partner’s medical care. Anyway, they are the experts. I was only informed that my partner was having a complicated labour and that she had to go for caesarean section and they asked me to sign the consent form for the operation.” (29-year-old driver)"

#### Reflection and resolution

The period after childbirth was a phase of reflection and resolution. The men referred to being present at the birth of their children as an opportunity to comprehend the childbirth process and resolve misconceptions they previously had. They stated that they were more knowledgeable about childbirth and appreciated the efforts of the hospitals and the midwives in encouraging male partner attendance during childbirth. The men recognized the important role of partner’s presence played in advocating for better care for their partners and newborn babies. One man reported that most of his and partner’s relatives lived in the northern part of the country making it impossible for them to accompany his partner during labour and birth. Thus, he became his partner’s labour companion, which he did not regret.

"“This is an excellent initiative for couples who do not have other female relatives at hand to support them. My partner was not left to labour alone. At least I was there by the bedside.”(35-year-old lawyer)."

The participants explained that their involvement in childbirth strengthened their relationship with their partners and children. They became very protective of their children and participated in childcare. The experience also increased their love and respect for their partners, and women in general. Most of the participants confirmed that the experience did not change their sex life although for some couples resumption of sexual activity took more than three months because of fear of hurting their partners, especially for those partners who had an episiotomy or caesarean section. Some of the men stated that having observed labour and birth, their desire to have more children diminished. Childbirth was perceived as putting the life of a partner at risk of death. They made unilateral decisions not to have any more children.

"“Giving birth is a life-threatening experience. I do not want her to go through that experience again. Better have one child with a mother than several without.” (39- year-old, programme coordinator)"

## Discussion

The demographic data of the participants in this study show that Malawian men who attended childbirth were educated and professional men. This group of men could be likened to middle-class men in the industrialized countries. Although it is not a tradition in Malawi for male partners to attend the birth of their children, the participants demonstrated that men can be labour companions for their partners just as women. Given an enabling environment, such as individual labour and childbirth rooms that provide privacy and pro-male partner labour companion policies, men can effectively assume the role of labour companions. Meerabeau stated that in the American society, the admission of male partners into the labour room was constructed as a public statement of family togetherness, a sign of a strong middle-class marriage [8]. However, this belief was not established in this study.

The findings of this study have illuminated on the importance of midwives, women and male peers as motivators for men to be labour companions. Male partner’s presence during labour and birth could contribute to continuous labour support in situations of midwives shortage. The role of midwives as educators and client advocates cannot be over emphasized. The midwives should take every opportunity to educate and advocate for male partner involvement in childbirth whenever possible.

Couple communication is vital for male partners’ attendance at childbirth. In this study, the couples discussed and made a joint decision on whether the male partner would be present. Feyisetan argued that spouse communication about reproductive health issues is greatly enhanced when both spouses have similar levels of education or close to one another [30]. He further argued that at higher levels of education and with little difference in educational attainment, partners appear to feel more comfortable discussing issues that were traditionally thought to be under the control of men [30].

The findings of this study have shown that informal male peer motivation moved some men to attend the birth of their children. Men who had attended the birth of their children tended to inform peers about their experiences and if positive, most likely encouraged them to participate. Avogo and Agadjanian found that men and women’s discussions in gendered networks are significantly associated with subsequent spouse communication in family planning [31]. They further asserted that social influence was directly reflected when informal social networks exchange information on childbearing [31].

The male partners in this study had multiple motives for attending the birth of their children. Whereas some of the participants stated that they attended the birth of their children out of curiosity, others did it to welcome their babies or be there for their partners. Unlike in the western society where prospective fathers are expected to attend and assist their partners at the time of childbirth [32], in Malawi men are not obliged to attend their children’s births. Their attendance at childbirth depends on among other things, the infrastructure, hospital policies, midwives’ attitudes towards men’s presence, and willingness of both the men and the expectant mothers. Nevertheless, midwives should endeavour to assist the male partners so that they should have an effective and positive experience.

Regardless of the motives to be present during childbirth, the men in this study assumed different roles including that of being their partner’s advocate and provider of psychological and emotional support. The men viewed being able to offer psychological and emotional support as positive experience. Somers-Smith stated that the mere presence of the partner makes the birthing woman feel valued, cared for, and appreciated [33]. Men’s presence during labour and childbirth has also been observed to reduce pain, anxiety, shorten the duration of labour, and less need for pain medication [33,34]. However, some studies have found less impact of partner’s attendance. For instance, Ip [35] found an increase in the amount of pain relieving drugs used and length of labour in women who had practical support from their partners. In addition, father’s support was not shown to reduce mother’s perinatal anxiety and pain [35]. Similar findings are also reported by Gungor and Beji in a study done in Turkey [15].

The negative experience of not being able to offer physical support to a partner in labour was attributed to lack of knowledge and skills related to unpreparedness. This finding confirmed previous findings [36]. Yardely asserted that fathers require information in order to provide the best support to their partners [37]. Longworth and Kingdon further suggested that if the male partner is more receptive to information on how to cope with labour and birth, he could relieve some of the pressure on both his partner and the health professional. They further expressed that an effective, positive birth scenario is one where the midwife, the woman and her partner work together to support each other [38].

The negative emotions of fear, anxiety, frustration and a sense of helplessness were also reported by other researchers [17,39,40]. According to a study conducted in South Africa, men reported fear of labour, operative interventions including episiotomy, not being a good father, and loss of marital closeness [41]. Chapman made a supposition that childbirth classes could possibly assist men in preparing for the changes they would witness in their partners during the labour experience [42]. Knowing that these changes are normal might reduce men’s levels of anxiety, frustration, and sense of helplessness [42].

However, it was not surprising to note that almost all of the men reported that they hid their negative emotions from their labouring wives. They pretended to be strong, as if all was well. In their study on men’s experiences with post-perinatal loss, O'Leary and Thorwick reported that fathers were reluctant to express fears because of the need to protect their partner [43]. According to Courtenay, men often viewed expressions of fear as a sign of weakness, and as such were reluctant to acknowledge it [44]. Nevertheless, health care providers should anticipate negative emotions from male partners and be able to assist them.

Lack of knowledge or preparation for labour, birth and their role in these processes contributed to men’s negative experiences. In turn, it created tension between the men and health care providers. Some of the men reported to be labelled ‘difficult’. Koppel and Kaise argued that hospital staff tended to underestimate and ignore the stress that fathers were going through when their partners were involved in an emergency birth [45]. Although Mapp and Hudson stated that during a stressful situation everyone including midwives and doctors, becomes stressed which makes communication difficult [46]. Nevertheless, the fathers who were prepared participate actively in the labour process, and their partners’ birthing-experiences tended to be better [47]. Even where fathers were minimally prepared, positive effect on the general experience for both men and women was observed [16,48].

Lack of involvement in decision making about the care of a spouse was perceived by the participants in this study as a negative experience. In most cases, women’s or couples’ input in the childbirth care was not sought. The women and their partners were just informed about the decisions made by the health care professionals. Consequently, the participants in this study perceived this behaviour by the health professions as a negative experience. Active involvement in decision-making by women and their male partners has been identified as an essential element of women-centred care [49,50]. This entails that women and their male partners are given adequate information so that they can make informed decisions that are best for them [51]. Therefore, maternity care providers should endeavour to provide opportunities for women and their male partners to be partners in decision-making. However, Dugas et al. asserted that coping with informed consumers in busy practice settings presents a challenge to health care providers who are accustomed to more paternalistic approaches [52].

The desire to have more children diminished in some of the men who attended the birth of their children. Similar results were reported by Carter in a study done in Guatemala [53]. However, there is need for further research to investigate the relationship between male partner’s attendance at childbirth and subsequent pregnancy intentions and practices.

### Limitations of the study

Recall bias could have been present even after restricting inclusion of men whose spouses delivered within the last 24 months prior to the study. Two years is a long period to remember much detail about one’s experiences. However, the inclusion period was extended to two years because of the scarceness of men who attended childbirth in the participating hospitals. The semi-structured approach coupled with in depth probing of the interviews helped to jog the memory of the participants and reduced recall bias.

Another limitation was that since the study population was urban, the findings may not be entirely applicable to other settings in Malawi. However, we gained insights into how similar populations can be targeted to improve male partner involvement during childbirth.

## Conclusions

From this study, it can be concluded that men’s preparation for attendance at labour and childbirth is a critical factor for a positive experience. Men may need education about labour and childbirth processes so that they are aware of what to expect when they accompany their partners for childbirth. This awareness may enable them to support their partners emotionally throughout the birthing process. The midwives have a duty to ensure that the emotional needs of their clients (the couples) are addressed. This practice could enhance a positive birthing experience for both women and their male partners. One of the midwifery practices that would help facilitate the positive birthing experience is to orient men to the routine care during labour and birth. This orientation would help mentally prepare them. The informational needs of male partners can be met through childbirth classes and also through easy-to-read materials. These adjuncts should be optimally used to help busy midwives better serve their clients.

## Abbreviations

ANC: Antenatal care; BAH: Blantyre Adventist Hospital; CHAM: Christian Hospital Association of Malawi; MCH: Maternal and Child Health; MMH: Mlambe Mission Hospital; MWK: Malawi Kwacha; NMTs: Nurse Midwifery Technicians; SDA: Seventh-Day Adventist; SRNMs: State Registered Nurse Midwives; USD: United States Dollar.

## Competing interests

The authors declare that they have no competing interests.

## Authors' contributions

LIK conceptualized the study, collected the data, led the analysis, and wrote the text of the paper. JS, EC and AM advised on the conceptualization of the study, analysis of the data, and presentation of the results. They also reviewed and edited the text. All authors read and approved the final manuscript.

## Pre-publication history

The pre-publication history for this paper can be accessed here:

http://www.biomedcentral.com/1471-2393/12/141/prepub

## Supplementary Material

Additional file 1 **Appendix1.** Interview guide. Click here for file
