# Supplementary material for: Malawian fathers’ views and experiences of attending the birth of their children: a qualitative study
Source: BMC Pregnancy Childbirth. 2012 Dec 5;12:141. doi: 10.1186/1471-2393-12-141 (PMC3520855; doi:10.1186/1471-2393-12-141)
Supplement: Additional file 1 — Appendix1. Interview guide. [file 1471-2393-12-141-S1.pdf]

**Appendix1: Interview guide**

**Malawian fathers' views and experiences of attending the birth of their child: A qualitative study**

Demographic data

Interviewer code__________

Note taker's code _________

Date ___________________

District____________________________ Location ____________________

Time: from_________________ to _____________

| | |
|---|---|
| Respondents code: | Age of the last born child |
| Address/Location | Age of the participant |
| Ethnicity | Marital status<br>(single) (divorced) (widow) (widower)<br>(co-habitating) |
| Educational level | Type of marriage<br>(monogamous) (polygamous) |
| Occupation | Number of children fathered |
| Partner's parity | Partner's mode of delivery (the labour and childbirth the man attended) |

**Guiding questions**

1. Can you tell me what motivated you to attend your partner's labour and childbirth?

   **Probe:**

   - Who motivated you?
   - What are the factors that motivated you to attend your partner's labour and childbirth?
   - How did you know that you can attend your partner's labour and childbirth?

2. Can you tell me in what aspects of your partner's labour and childbirth care did you participate?

   **Probe**

   - Can you tell me how you participated in the aspects of care you have mentioned? (*Make a list of the aspects of care participated in, and mention one by one for the participant to explain how he participated).*

3. Can you tell me the experiences you had when you attended your partner's labour and childbirth?

   **Probe**

   - Can you tell me more about your positive experiences during labour?
   - Can you tell me more about your negative experiences during labour?
   - What do you think contributed to the positive experiences you had?
   - What do you think contributed to the negative experiences you had?
   - Can you tell me how you reacted to the positive experiences you had?
   - Can you tell me how you reacted to the negative experiences you had?
   - What do you think could be done to enhance the positive experiences?
   - What do you think could be done to minimize the negative experiences?
